# Review of, "Biostatistics and Epidemiology" by S. Wassertheil-Smoller

**DOI:** 10.1186/1475-925X-3-36

**Published:** 2004-10-19

**Authors:** Daniel J Schneck

**Affiliations:** 1 Virginia Tech, Blacksburg, Virginia 24061-0219, USA

If you need to know anything *basic *about Biostatistics and Epidemiology, this book *belongs *in your library! Unlike most science and technology books – which, I have concluded after nearly 42 years in this business, tend to be written less to teach and communicate information than to impress the reader with how much the *author *knows about the subject matter – this one, refreshingly and quite successfully, ensures that the *reader *comes away with a better understanding of the topics of Biostatistics and Epidemiology, as a result of the authors' genuine desire to convey information in a clear, concise, relevant, understandable, and very readable way.

In nine Chapters and nine Appendices, spanning 243 pages (which include ample references and suggested further readings), the indexed material covers a wide range of fundamental principles that form the basis for such subjects as: (1) The Scientific Method; (2) Probability Theory; (3) Statistics; (4) Epidemiology; (5) Screening Methods and Techniques; (6) Clinical Trials; (7) Quality of Life; (8) Genetics; and (9) Biomedical Ethics. A unique feature of the way the book is written, is that individual Chapters can be read out-of-sequence with no loss of continuity. The reader can skip around, omit certain sections, and still glean what he or she needs to know without feeling cheated. That's not easy to do, but this author does it well.

I found the material to be written at a very comfortable cognitive level – aimed mainly at medical, upper-level-college, and graduate students – and sequenced in a logical manner, thus making it easy to follow and totally user-friendly. The author has a special talent for reducing complicated concepts to an easily understandable level. For example the Appendix dealing with Genetic Principles gives the best introductory synopsis of this topic that I have seen anywhere; and the "middle Chapters" that address "Mostly About" – Statistics (Chapter 3), Epidemiology (Chapter 4), Screening (Chapter 5), Clinical Trials (Chapter 6), Quality of Life (Chapter 7), and Epidemiology (Chapter 8), are gems!.

What are especially nice are the many relevant, easy-to-understand and practical examples, that the author judiciously and effectively intersperses with the theoretical background material; and, the Chapter Summaries that conclude most of them. There is a very well-balanced give-and-take between theory and practice. I also liked the wonderful (sometimes witty) little parables and anecdotes that give the book a charming personality. In all, I would offer my compliments to the author for a "job well done!"

